# Combination immunotherapy and anti-angiogenic therapy shows promising efficacy in NSCLC patients with recurrent or refractory brain metastases and negative driver genes

**DOI:** 10.3389/fimmu.2025.1684759

**Published:** 2025-12-03

**Authors:** Liwei Sun, Bonian Chen, Bin Wang, Jinduo Li, Lin Li, Caijuan Tian, Hongbo Zhang, Pengfei Liu, Xiaomin Liu

**Affiliations:** 1 Department of Oncology, Tianjin Huanhu Hospital, Tianjin Key Laboratory of Cerebral Vascular and Neurodegenerative Disease, Tianjin Neurosurgical Institute, Tianjin, China; 2 Department of Medical Insurance, Tianjin Huanhu Hospital, Tianjin Key Laboratory of Cerebral Vascular and Neurodegenerative Disease, Tianjin Neurosurgical Institute, Tianjin, China; 3 Tianjin Marvel Medical Laboratory, Tianjin Marvelbio Technology Co., Ltd, Tianjin, China; 4 Department of Oncology, Institute of Basic Research, Tianjin Academy of Traditional Chinese Medicine, Tianjin Academy of Traditional Chinese Medicine Affiliated Hospital, Tianjin, China

**Keywords:** prospective observational study, non-small cell lung cancer, brain metastasis, immunotherapy, anti-angiogenic therapy

## Abstract

**Introduction:**

This study aimed to evaluate the efficacy of immunotherapy combined with anti-angiogenic therapy for non-small cell lung cancer (NSCLC) with brain metastasis (BM) and negative driver genes.

**Methods:**

This observational prospective study was conducted using the clinical data of 34 patients with NSCLC and BM, including 24 patients who received immunotherapy combined with anti-angiogenic therapy and 10 control patients using oral anlotinib upon the first- to fourth-line treatment failure or refusing to continue chemotherapy between June 2017 and August 2022. Efficacy was evaluated using progression-free survival (PFS), objective response rate (ORR), disease control rate (DCR), and adverse reactions.

**Results:**

Among 24 patients treated with immunotherapy combined with anti-angiogenic therapy, 17 had a partial response, six had stable disease, and one had progressive disease. The ORR and DCR of patients receiving immunotherapy combined with anti-angiogenic therapy were 70.8% and 95.8%, respectively. The median PFS of immunotherapy combined with anti-angiogenic treatment was significantly longer than that of oral anlotinib (18.0 *vs*. 4 months, log-rank test, p < 0.0001), indicating that immunotherapy combined with anti-angiogenic therapy can substantially improve the treatment efficacy for NSCLC with BM and negative driver genes. Adverse reactions mainly included rash in one case and hypothyroidism in one case, but did not involve myocardial damage or liver and kidney function damage.

**Conclusion:**

Immunotherapy combined with anti-angiogenic therapy in patients with recurrent or refractory NSCLC and BM driven by negative genes has yielded promising but preliminary findings.

**Clinical Trial Registration:**

www.chictr.org.cn, identifier ChiCTR1800017499

## 1 Introduction

Lung cancer remains the leading cause of cancer-related morbidity and mortality in China (1, 2). Brain metastases (BMs) are associated with significant morbidity and occur in approximately 50% of patients with advanced non-small cell lung cancer (NSCLC) (3). Untreated metastatic lesions may result in death within 1–2 months (4). Treatment options for lung cancer with BM include surgery, chemotherapy, whole-brain radiotherapy, stereotactic radiotherapy, molecular targeted therapy, and immunotherapy (5). Although whole-brain radiation therapy (WBRT) could prolong the median survival of patients with NSCLC and BM by approximately 4 months (6), its associated fatigue and neurocognitive toxicity could affect patients’ acceptance of the therapy (7). Cytotoxic chemotherapy is considered for the treatment of multiple metastases in patients with NSCLC (8). Traditionally, chemotherapeutic drugs face great challenges in crossing the blood–brain barrier, resulting in limited effectiveness in treating NSCLC with BM. Recent studies have indicated that the blood–brain barrier can be disrupted by decreasing Msfd2 expression to promote docosahexaenoic acid transport and lipid metabolism through tumor metastatic lesions (9), indirectly facilitating the penetration of chemotherapeutic drugs (10). Low-dose anti-angiogenic drugs can rescue abnormal vascular tumors, improve hypoxia, and activate immune cells (11). Most studies have reported that platinum-based chemotherapy combined with pemetrexed or docetaxel is an effective first-line treatment for non-squamous NSCLC with BM (12–15).

The 2016 National Comprehensive Cancer Network (NCCN) treatment guidelines for NSCLC recommend angiogenesis inhibitors combined with chemotherapeutic drugs as the first-line standard treatment. The 2021 NCCN treatment guidelines for NSCLC state that immunotherapy, with or without chemotherapy, is recommended for patients who do not have actionable molecular biomarkers (16). Moreover, the NCCN guidelines for NSCLC indicated that atezolizumab and pembrolizumab, as immunotherapy, showed excellent efficacy for programmed cell death ligand 1 (PD-L1) ≥1% and PD-L1 ≥50%, respectively. KEYNOTE-042 clinical trials demonstrated that first-line pembrolizumab monotherapy harbored longer progression-free survival (PFS) than chemotherapy for patients with untreated metastatic NSCLC and PD-L1 tumor proportion scores ≥50%, ≥20%, and ≥1% (17). However, the benefit of adjuvant immunotherapy in patients with PD-L1<1% remains unclear (18).

Clinical studies have demonstrated that immunotherapy combined with other therapies shows longer PFS [hazard ratio (HR) = 0.48, 95% confidence interval (CI): 0.41–0.56] and overall survival (OS) (HR = 0.64, 95% CI: 0.60–0.69) compared to immunotherapy alone (19). Although the upregulation of immune checkpoints is associated with tumor-induced angiogenesis, disrupting the tumor crosstalk network can enhance immune-driven protection (20). Clinical developments of angiogenesis blockade therapy have focused on vascular endothelial growth factor (VEGF), which relies on impairing the vascular network to eliminate the nutrient and oxygen supply to malignant cells and is usually combined with immunotherapy and chemoradiotherapy (21). Anlotinib, a broad spectrum of inhibitors of tumor angiogenesis, has longer PFS (median, 4.17 *vs*. 1.30 months) and OS (median, 8.57 *vs*. 4.55 months) than placebo for patients with NSCLC and BM, indicating high potential to manage the intracranial lesions (22). Pembrolizumab combined with bevacizumab for the treatment of patients with stage IV lung adenocarcinoma with negative driver genes can improve the efficacy, immune function, and PFS (23). Sintilimab (a PD-1 antibody) and bevacizumab therapy for intermediate-stage hepatocellular carcinoma are effective, safe, and inexpensive in China (24, 25). Moreover, Zhang et al. (26) reported that sintilimab plus docetaxel as second-line therapy of advanced NSCLC without targetable mutation achieved median PFS of 4.5 and 2.6 months for PD-L1 (<1%)/CD8 (≥1%) and PD-L1 (<1%)/CD8 (<1%), respectively. Immunotherapy combined with anti-angiogenic therapy is an alternative option for patients with advanced NSCLC; however, most trials may exclude patients with brain metastases, limiting the generalizability of the results. A randomized, double-blind, multicenter, phase III trial (NCT0380224) indicated that sintilimab plus bevacizumab and chemotherapy for treating NSCLC achieved better clinical outcomes than chemotherapy alone (median PFS, 6.9 *vs*. 4.3 months) (27). Adverse events include decreased neutrophil count, decreased blood cell count, and anemia (27). A phase II clinical trial (NCT04213170) that observed intracranial objective response rate (ORR), intracranial PFS, PFS, and ORR to evaluate the treatment effect of sintilimab combined with bevacizumab for NSCLC with BM remains underway.

Based on the histopathological results, 34 patients with stage IV NSCLC adenocarcinoma with BM and negative driver genes between June 2017 and August 2022 were enrolled. After first-line therapy with platinum-based chemotherapy, immunotherapy (sintilimab) combined with anti-angiogenic therapy (bevacizumab or anlotinib) or oral anlotinib was conducted to treat patients who refused to continue chemotherapy. The efficacy evaluation aimed to confirm the benefit of immunotherapy combined with anti-angiogenic therapy for NSCLC with BM, negative driver genes, and PD-L1 < 1%. PD-L1 expression was evaluated on tumor tissue using immunohistochemistry (IHC). All patients were tested using the same standardized assay and scoring system.

## 2 Materials and methods

### 2.1 Patients

This single-center clinical trial was conducted on the clinicopathological features, therapeutic regimens, and effects in 40 patients with NSCLC and BM and negative driver genes in our hospital between 2017 and 2022. Five patients who withdrew from the trial midway and one patient with squamous cell carcinoma were excluded. Therefore, 34 patients were enrolled: 24 in the immunotherapy combined with anti-angiogenic therapy group and 10 patients in the oral anlotinib group (**Table 1**, **Supplementary Tables S1- S3**).

**Table 1:** Basic information of patients.

Characteristics	Immunotherapy combined with anti-angiogenic therapy group	Control group (oral anlotinib)	p-Value
Totals	24	10	
Gender, n (%)			0.692
Male	17 (70.83%)	6 (60.00%)	
Female	7 (29.17%)	4 (40.00%)	
Age (years), median (± Interquartile Range" (IQR))	63.5 (57.5–67.0)	69.0 (62.0–70.0)	0.079
Adenocarcinoma	24 (100%)	10 (100%)	–
American Joint Committee on Cancer (AJCC) TNM stage			–
Stage IV	24 (100%)	10 (100%)	
Neurologic symptoms			0.109
Headache or dizziness	15 (62.5%)	4 (40.0%)	
Epilepsy	2 (8.33%)	2 (20.0%)	
Weakness in the lower limbs, limb numbness, or limb movement disorder	2 (8.33%)	4 (40.0%)	
Other symptoms	3 (12.50%)	–	
No symptoms	2 (8.33%)	–	
Number of BMs, mean (95% CI)	5.83 (4.17–7.53)	6.30 (3.48–9.12)	0.758
Volume of largest BMs (cm^3^), mean (95% CI)	7.38 (5.02–9.73)	3.17 (2.60–3.75)	0.037
Mean arterial pressure (mmHg), mean (95% CI)	101 (98.94–103.34)	99.23 mmHg	0.558
Fasting blood glucose (mmol/L), mean (95% CI)	5.46 (5.09–5.82)	5.65 (5.06–6.24)	0.570
Red blood cell count (×10^9^/L), mean (95% CI)	3.99 (3.80–4.18)	3.93 (3.68–4.18)	0.732
White blood cell count (×10^9^/L), mean (95% CI)	5.78 (5.23–6.33)	5.28 (4.48–6.08)	0.318
Neutrophil count (×10^9^/L), mean (95% CI)	3.61 (3.15–4.08)	3.13 (2.61–3.65)	0.234
PD-L1 tumor proportion score			1.000
<1%	22 (91.67%)	10 (100%)	
1%–50%	2 (8.33%)	0 (0%)	
>50%	0 (0%)	0 (0%)	
Therapy			0.018
Second-line therapy	9 (37.50%)	0 (0%)	
Third-line therapy	14 (58.33%)	6 (60.00%)	
Fourth-line therapy	1 (4.17%)	4 (40.00%)	

BMs, brain metastases.

The inclusion criteria were as follows:

1. Aged ≥18 years.
2. Histologically confirmed stage IV recurrent or refractory NSCLC.
3. Pathology, puncture, and biopsy indicating an adenocarcinoma.
4. Head magnetic resonance imaging (MRI), chest computed tomography (CT), or positron emission tomography (PET)–CT imaging results indicate multiple metastatic lesions.
5. Negative driver genes (including EGFR mutations, ALK fusion, and ROS1 fusion), negative driver genes (EGFR, ALK, and ROS1) confirmed by next-generation sequencing (NGS) panel.
6. PD-L1 tumor proportion score <1%.
7. Patients have not used immunosuppressive agents in the previous treatment.
8. Estimated survival period is >3 months.
9. Patients agreed to participate in the study and provided signed informed consent.

The exclusion criteria were as follows:

1. Patients having pulmonary fibrosis or other malignant tumors.
2. Patients suffering from autoimmune or severe cardiovascular diseases.
3. Having a history of infusion reactions for antibody therapy.
4. Patients undergoing radiotherapy for NSCLC with BM.
5. Other factors that should be ruled out.

Twenty-four patients were treated with immunotherapy plus anti-angiogenic therapy, and 10 patients received oral anlotinib after first-line platinum-based or multi-cycle chemotherapy if they refused to continue chemotherapy and selected an alternative treatment because of the therapeutic effect (**Supplementary Tables S2**, **S3**). The single-center study evaluating cerebrospinal fluid and brain metastatic lesions in patients with lung cancer brain metastases was prospectively registered in the Chinese Clinical Trial Registry (ChiCTR1800017499) in 2018. Plasma and CSF samples were drawn from consenting NSCLC patients with diagnosed brain metastasis. All patients had no driver gene mutation and were diagnosed with NSCLC adenocarcinoma by histopathology or puncture biopsy. Metastatic lesions were screened using head MRI (for the brain), chest CT, or PET–CT. All patients had advanced (stage IV), recurrent, and refractory NSCLC with BM. The main reasons for admission were intermittent headache, dizziness, cough, facial nerve palsy, chest tightness, and language impairment. The time to symptom remission in the 24 patients treated with immunotherapy combined with anti-angiogenic therapy differed.

### 2.2 Treatment methods

**Figure 1:**
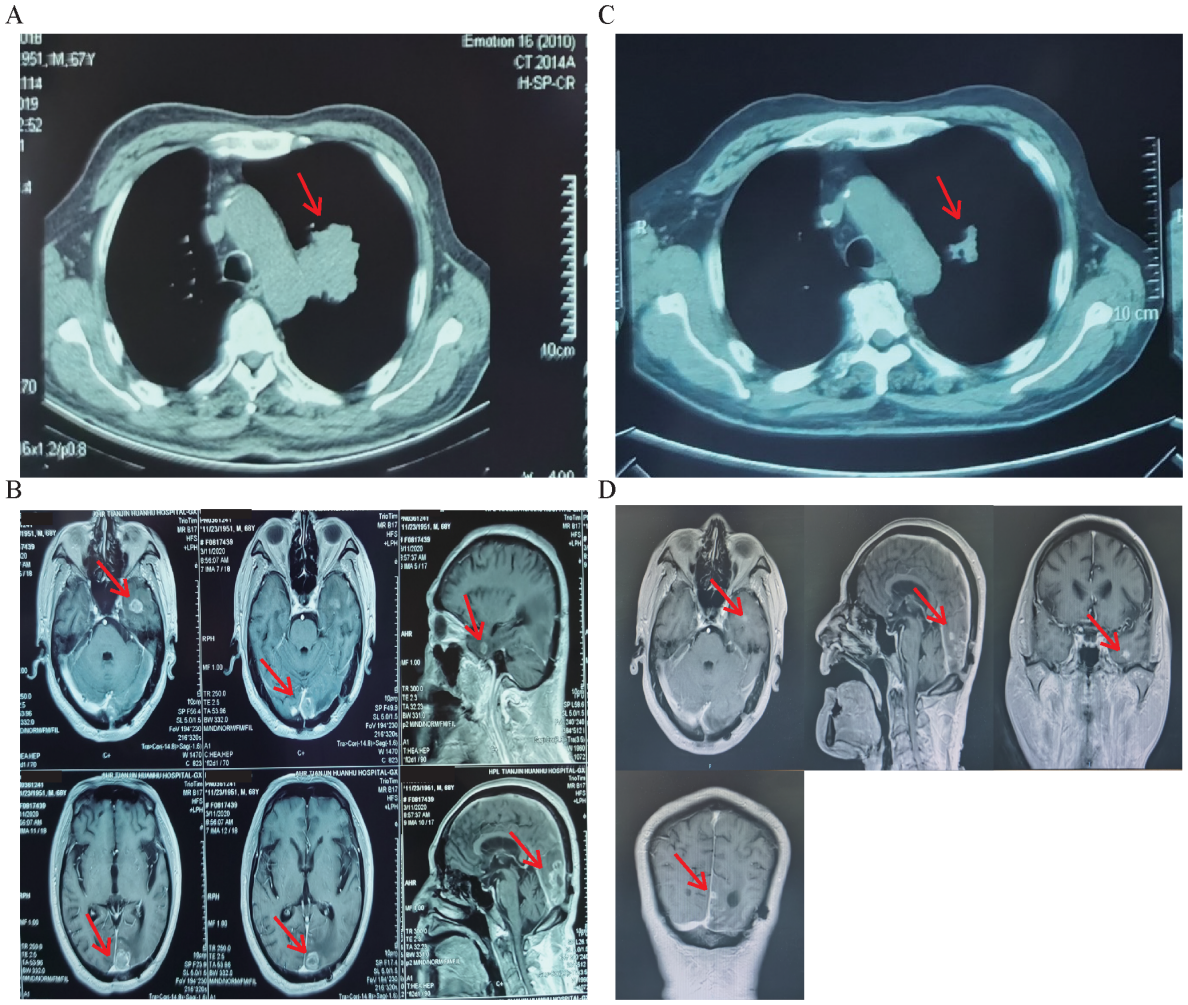
Chest computed tomography (CT) and head magnetic resonance imaging (MRI) results of case 2. **(A, B)** Chest CT and MRI results before immunotherapy combined with anti-angiogenic therapy. **(C, D)** Chest CT and MRI results after three cycles of immunotherapy combined with anti-angiogenic therapy. Arrows point to the location of the lesion.

**Figure 2:**
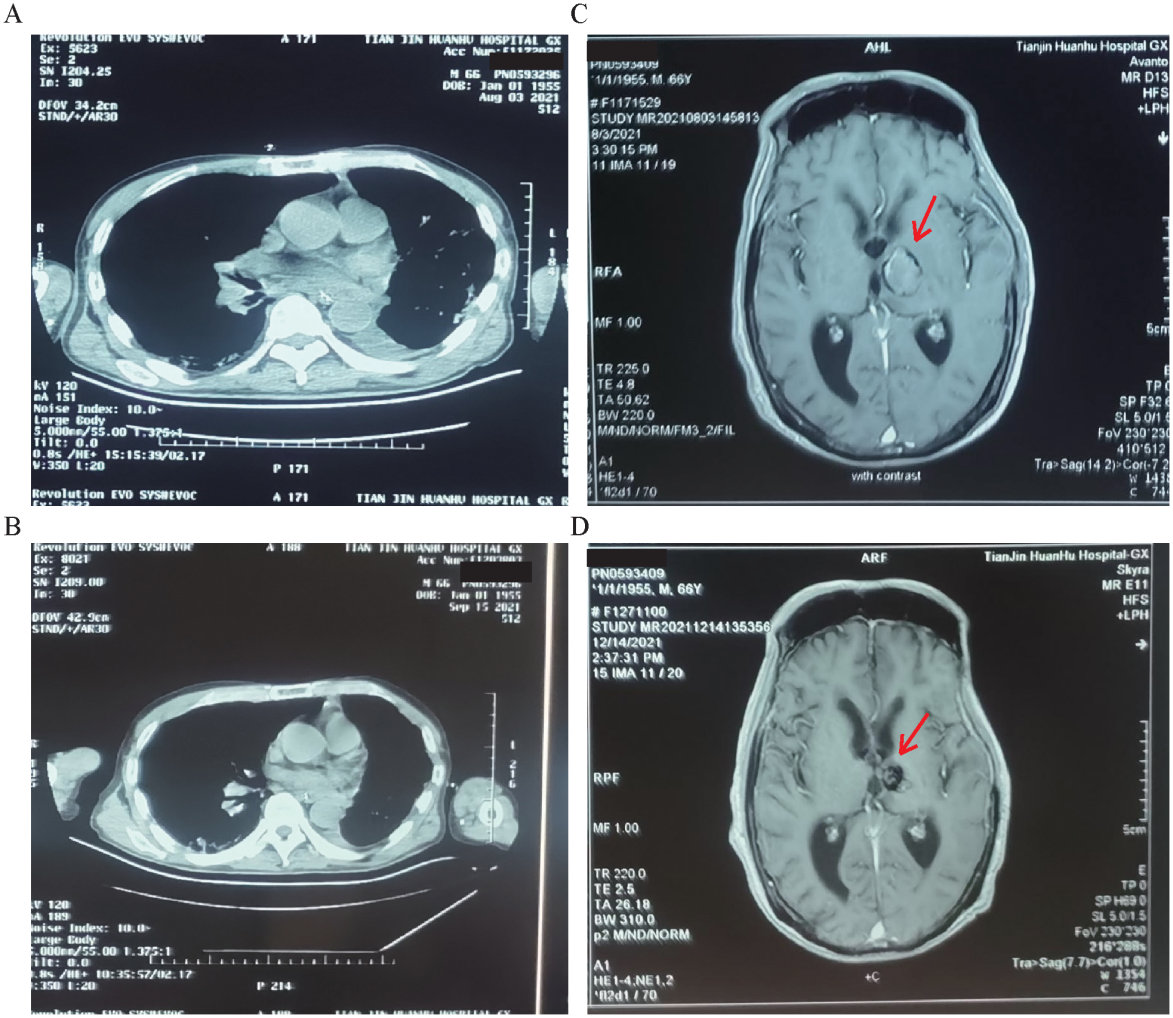
Chest CT and head MRI results of case 11. **(A, B)** Chest CT and MRI results before immunotherapy combined with anti-angiogenic therapy. **(C, D)** Chest CT and MRI results after four cycles of immunotherapy combined with anti-angiogenic therapy. Arrows point to the location of the lesion.

Platinum-based chemotherapy was used in all patients as the first-line therapy for NSCLC with BM. After the first- to fourth-line treatment failed or refusal to continue chemotherapy, the curative effect of immunotherapy combined with anti-angiogenic therapy on lung and intracranial tumors was analyzed. For the group receiving immunotherapies combined with anti-angiogenic therapy, sintilimab should be administered, followed by bevacizumab after at least 5 min; both administrations should be completed on the same day. Sintilimab (200 mg) was administered via intravenous infusion every 3 weeks, with each 3-week period considered as one cycle. Bevacizumab (10–15 mg/kg) was administered via intravenous infusion every 3 weeks. The combined infusion should not exceed six cycles (equivalent to 18 weeks) or should be discontinued if the patient experiences disease progression or intolerance. As 17 patients were still being followed up, efficacy evaluation, PFS, ORR, disease control rate (DCR), and adverse reactions were evaluated for the immunization combined with anti-angiogenic therapy group and the control group. The control group was administered anlotinib orally at a dose of 12 mg or an adjusted dose of 10 mg once daily for two consecutive weeks, followed by a 1-week break. Each cycle lasted 3 weeks and continued until disease progression or occurrence of intolerable adverse reactions. Chest CT and head MRI of two representative cases before and after immunization combined with anti-angiogenic therapy were collected (**Figures 1**, **2**).

### 2.3 Statistical analysis

All statistical analyses were performed using SPSS 27.0 and GraphPad Prism 9.0. Continuous variables were expressed as mean ± standard deviation or median, depending on data distribution; categorical variables were presented as counts and percentages. PFS was calculated from the start of immunotherapy or anlotinib treatment until documented disease progression or death from any cause. PFS curves were plotted using the Kaplan–Meier method, and differences between groups were compared using the log-rank test. All tests were two-sided, and a p-value <0.05 was considered statistically significant.

## 3 Results

### 3.1 Clinicopathological features

Thirty-four cases were adenocarcinomas. One patient with squamous cell carcinoma was excluded owing to bevacizumab only for non-squamous NSCLC. There were no significant differences between the two groups regarding baseline data or symptoms (**Table 1**). However, the combination therapy group had a significantly larger tumor volume and longer mean PFS in the third- and fourth-line treatments than the oral anlotinib group (**Table 1**, **Supplementary Tables S2**, **S3**). In the immunotherapy combined with anti-angiogenic therapy group, 17 patients were still being followed up. We admitted PD-L1 20% and PD-L1 10% patients (**Supplementary Tables S1**, **S2**). To assess the potential impact of these patients on our results, we performed a sensitivity analysis excluding them. After exclusion, the median PFS was 14 months in the treatment group and 4 months in the control group, consistent with the overall analysis (Student’s t-test, p-value <0.05). These findings indicate that the inclusion of the two patients with PD-L1 >1% did not materially affect the main conclusions. Case 11 showed intracranial metastatic lesions that significantly diminished after using sintilimab plus bevacizumab for treatment (**Figure 2**), and PFS was 20 months, slightly longer than the median PFS; case 28 was still being followed up (PFS >17 months). The main causes of admission were intermittent headache, dizziness or cough, crooked mouth, disturbance of consciousness, and chest stuffiness. All 34 cases had negative driver genes. The time to symptom remission in the immunotherapy combined with anti-angiogenic therapy group differed.

### 3.2 The treatment process

Patients with NSCLC and BM received immunotherapy combined with anti-angiogenic therapy after failure of first- to fourth-line therapy. In the immunotherapy combined with anti-angiogenic therapy group, second-, third-, and fourth-line therapies were administered in nine, 14, and one patient, respectively. Of the patients, 62.5% received third-line or more therapies. After multi-cycle chemotherapy and immunotherapy combined with anti-angiogenic therapy, especially after multiple chemotherapies, some patients experienced side effects and refused to continue chemotherapy. This led to the choice of alternative treatments. In the control group, four patients received fourth-line therapy, and six patients received third-line therapy.

### 3.3 The treatment effect

**Figure 3:**
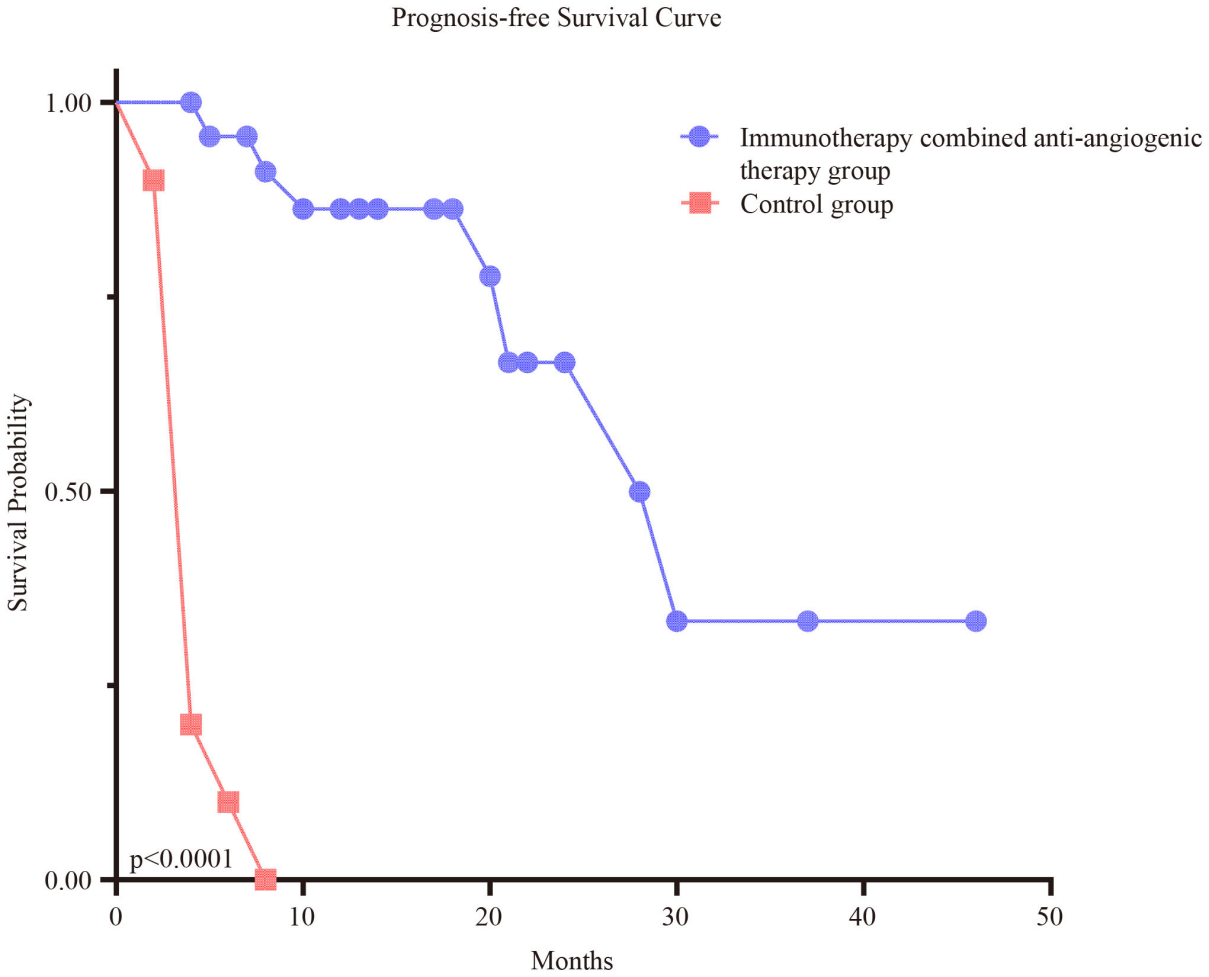
The median PFS in the immunotherapy combined with anti-angiogenic therapy group was significantly longer than in the control group. The blue circle line represents the patients treated with immunotherapy combined with anti-angiogenic therapy, while the orange rectangle line represents the patients treated with oral anlotinib. PFS, progression-free survival.

All 24 patients had relapsed or refractory NSCLC and BM, with negative driver gene expression. After multi-cycle chemotherapy and an immunotherapy combined with anti-angiogenic therapy, the patients’ conditions progressed. Immunotherapy (sintilimab 200 mg) combined with anti-angiogenic drug therapy (bevacizumab 10 or 15 mg/kg) was administered once every 3 weeks. Among the 24 patients, 17 had a partial response (PR), six had stable disease (SD), and one had progressive disease (PD). Currently, 17 patients are still being followed up, and 17 patients have died. The ORR and DCR values were 70.8% and 95.8%, respectively. The median PFS was significantly longer in the immunotherapy combined with anti-angiogenic therapy group compared to the oral anlotinib group (18.0 [95% CI: 14.0–20.0] *vs*. 4.0 months [95% CI: 4.0–5.0]), HR = 0.1440 [95% CI: 0.02297–0.8954] (log-rank test, p < 0.0001). As shown in the Kaplan–Meier curve (**Supplementary Figure S1**), patients receiving immunotherapy combined with anti-angiogenic therapy exhibited improved PFS compared with oral anlotinib (log-rank test, p = 0.0178). Patients achieving PR or SD exhibited significantly longer median survival than the oral anlotinib group (**Figure 3**). Immunotherapy combined with anti-angiogenic therapy showed longer mean PFS than oral anlotinib (**Supplementary Tables S2**, **S3**). Due to the limited sample size, particularly in the control group (n = 10), the statistical power of subgroup comparisons may be restricted. Therefore, larger multicenter studies with an expanded patient cohort are warranted in the future to further validate our conclusions.

Chest CT and head MRI results of cases 2 and 11 before and after immunotherapy (sintilimab) combined with anti-angiogenic therapy (bevacizumab) are shown in **Figures 1** and **2**, respectively. In case 2, after treatment with sintilimab plus bevacizumab, pulmonary and brain metastatic lesions were diminished based on chest CT and head MRI imaging results (**Figure 1**). The PFS in case 11 was 20 months, although PD-L1 expression was 20%. After immunotherapy combined with anti-angiogenic therapy, the efficacy evaluation of cases 2 and 11 showed PR without adverse reactions.

### 3.4 The occurrence of adverse reactions

Adverse events (AEs) were monitored throughout treatment via clinical examination, laboratory tests (blood counts, and liver/renal and thyroid function), imaging (chest CT, head MRI, or PET–CT), and patient-reported symptoms. Treatment-related adverse reactions were generally mild. In the combination therapy group, adverse reactions included rash in one patient and hypothyroidism in one patient, both graded as Common Terminology Criteria for Adverse Events (CTCAE) 1–2. No Grade ≥ 3 adverse events were observed (**Supplementary Table S4**). No immune-related adverse events (irAEs) occurred during the study period.

## 4 Discussion

Common metastatic sites of lung cancer include intracranial, intrapulmonary, liver, and bone metastases. The presence of distant metastasis indicates advanced NSCLC. According to Li et al., NSCLC patients without driver gene alterations benefit most from combined chemotherapy and radiotherapy with the addition of anti-angiogenic therapy or immunotherapy (28). Notably, Shan et al. reported that two patients with advanced NSCLC adenocarcinoma lacking driver gene alterations maintained durable responses after discontinuing immune checkpoint inhibitors, with one achieving Complete Response (CR) and PFS > 30 months, and the other achieving PR and PFS > 9 months (29). ECOG4599 and BEYOND, phase III randomized controlled studies, and a recent meta-analysis indicated that platinum-based chemotherapy combined with bevacizumab as the first-line treatment of advanced non-squamous NSCLC significantly prolonged the OS and PFS of patients compared with chemotherapy alone (30). A multicohort phase I study evaluated the anti-tumor activity and safety of ramucirumab combined with pembrolizumab in multiple solid tumors, including 27 patients with previously treated advanced NSCLC. The disease control rate reached 86%, with a median PFS and OS of 9.7 and 26.2 months, respectively (31). Similarly, a phase I trial showed antitumor activity in a cohort of heavily pre-treated NSCLC patients using ramucirumab plus durvalumab, with median PFS and OS of 1.7 and 12.4 months, respectively (32). In a phase II trial of anti-PD-1 induction therapy for unresectable stage III NSCLC, 55.3% of patients with negative PD-L1 expression achieved partial response, 31.9% had stable disease, and 2.1% progressed, demonstrating the potential efficacy of PD-1-based therapy regardless of PD-L1 expression (33). It has been reported that adding anti-angiogenic therapy to cranial radiotherapy and PD-1 inhibitors significantly improves intracranial local and distant progression-free survival in driver gene-negative NSCLC patients with brain metastases, with manageable toxicity (34). Many clinical trials have shown that anti-cancer efficacy is improved and that survival is prolonged after adding anti-angiogenic agents to Immune Checkpoint Inhibitors (ICIs) (35). Here, among the 24 patients, 17 had PR, six had SD, and one had PD. The ORR and DCR values were 70.8% and 95.8%, respectively. The median PFS of the immunotherapy combined with anti-angiogenic therapy group was 18 months (95% CI: 14.0–20.0), which was significantly higher compared with that of the oral anlotinib group (median PFS, 4 months [95% CI: 4.0–5.0]) (log-rank test, p < 0.001). We observed a significant reduction in lung and brain lesions in case 2 after immunotherapy combined with anti-angiogenic therapy (**Figure 1**). Although we have clinically proven that immunotherapy combined with anti-angiogenic therapy achieved good results in treating NSCLC with BM, the study’s sample size was small, and further case collection is needed.

In the era of evidence-based medicine, patients should be informed of the evidence gap, offered opportunities to carefully discuss the advantages and disadvantages of all possible therapeutic schedules, and empowered to make decisions (36). Chemotherapy regimens generate different side effects. One study aimed to achieve better treatment outcomes by making patient-centered choices and patient-preferred side effects (37). In a systematic review and meta-analysis, Chai et al. (38) reported that immunotherapy combined with chemotherapy to treat PD-L1 and driver gene-negative advanced NSCLC was significantly superior to chemotherapy alone regarding ORR, OS, and PFS. Side effects during tumor chemotherapy differ individually, and corresponding coping methods should be adopted according to reactions, such as paying attention to diet, taking drugs that can reduce chemotherapy side effects, and changing treatment methods.

ICIs, such as anti-cytotoxic T lymphocyte antigen 4 (CTLA-4), anti-programmed cell death 1, and anti-programmed cell death 1 ligand 1 antibodies, have demonstrated the most significant advances in oncotherapy over the last decade (39). The incidence of ICI-related fatal adverse events is approximately 0.3%–1.3% (40). This risk remains lower than that associated with conventional therapies: 0.9% with platinum-based double-drug chemotherapy (41), 15% with allogeneic hematopoietic stem cell transplantation (39), and 0%–4% with targeted therapies, such as VEGF-targeted drugs or tyrosine kinase inhibitors (TKIs) (42). Immunotherapy-associated adverse events associated with anti-PD-1 antibodies are less frequent and have a different spectrum of organ involvement than anti-CTLA-4 antibodies (39). Additionally, sintilimab combined with bevacizumab and radiotherapy is considered a safe and effective regimen for first-line treatment of hepatocellular carcinoma, with no treatment-related deaths reported (43). The safety data for bevacizumab in NSCLC presented in the recent AVAiL trial indicate that, similar to clinical trials with other indications, adverse events can generally be controlled using standard clinical techniques, and discontinuation of bevacizumab treatment is uncommon (44). Anti-angiogenic therapy can normalize tumor vasculature and reshape the tumor microenvironment, enhancing immune cell infiltration and function, thereby providing a critical biological rationale for its synergy with immune checkpoint inhibitors to improve antitumor efficacy (45). Theoretically, anti-angiogenic agents can promote tumor vessel normalization, facilitating T-cell infiltration and drug delivery to the tumor. For combination therapy, a lower dose of ICI is adequate to resist an immunosuppressive microenvironment with a lower incidence of adverse events (46).

Many preclinical studies have demonstrated the possible mechanisms of abnormal angiogenesis in the adjustment of antitumor immunity in mouse tumor models and favor combining immunotherapy and anti-angiogenesis for cancer therapies (47). Sintilimab plus bevacizumab biosimilar IB1305 plus chemotherapy for patients with EGFR-mutated NSCLC showed longer median PFS than the sintilimab plus chemotherapy and chemotherapy alone groups (median PFS, 9.8, *vs*. 6.9, *vs*. 4.3 months) (27). Preliminary data from several ongoing clinical trials indicate that this combination therapy is well-tolerated (48). However, because of the complex regulatory mechanisms of these two types of therapies, ways to collaboratively apply them to achieve the optimal treatment outcome remain unknown, including identifying biomarkers with accurate responses to the combination therapy and optimizing the drug dosage, dosage regimen, and treatment timing (47). Here, 24 patients received immunotherapy (sintilimab 200 mg) combined with anti-angiogenic therapy (bevacizumab 10 or 15 mg/kg) once every 3 weeks after failure of the first- to fourth-line therapy. Among them, 62.5% received third-line or more treatments. The ORR and DCR values were 70.8% and 95.8%, respectively. The median PFS was prolonged by 14 months, which was significantly longer than that in the control group. To receive approval for first-line lung cancer treatment, translational research and innovative clinical trials, such as optimizing the drug dose, drug delivery strategies, and the method and timing of administration, are needed.

Combining immunotherapy with anti-angiogenic therapy in patients with recurrent or refractory BM from NSCLC with negative driver genes yielded promising results regardless of PD-L1 expression. Further clinical studies are required to investigate the exact therapeutic effects.

Despite the promising clinical outcomes observed, this study has several limitations that should be acknowledged. First, this was a single-center prospective observational study with a small sample size, which may limit the generalizability and statistical power of our findings. Second, a key limitation of this study is that OS data remain immature due to ongoing follow-up. Longer observation is required to assess OS benefit, which will be an important endpoint in our subsequent analyses. Third, treatment allocation was not randomized, and the control group included patients who refused chemotherapy. This may lead to self-selection bias, as the decision to decline chemotherapy could be associated with factors such as Eastern Cooperative OncologyGroup (ECOG) performance status, disease burden, socioeconomic conditions, or personal treatment preferences, potentially compromising between-group comparability. Although this bias is unavoidable in the current study design, future research could mitigate this through statistical approaches such as propensity score matching (PSM) or multivariate Cox regression analysis to better balance baseline differences and reduce confounding effects.

## Data Availability

The original contributions presented in the study are included in the article/**Supplementary Material**. Further inquiries can be directed to the corresponding authors.

## Supplementary material

Supplementary Figure 1 Kaplan-Meier survival curves depicting progression-free survival for all patients in the immunotherapy combined with anti-angiogenic therapy group versus the control group (Log-Rank test, p < 0.05).
