# Supplementary figures and images for: Combination immunotherapy and anti-angiogenic therapy shows promising efficacy in NSCLC patients with recurrent or refractory brain metastases and negative driver genes
Source: Front Immunol. 2025 Dec 3;16:1684759. doi: 10.3389/fimmu.2025.1684759 (PMC12708319; doi:10.3389/fimmu.2025.1684759)

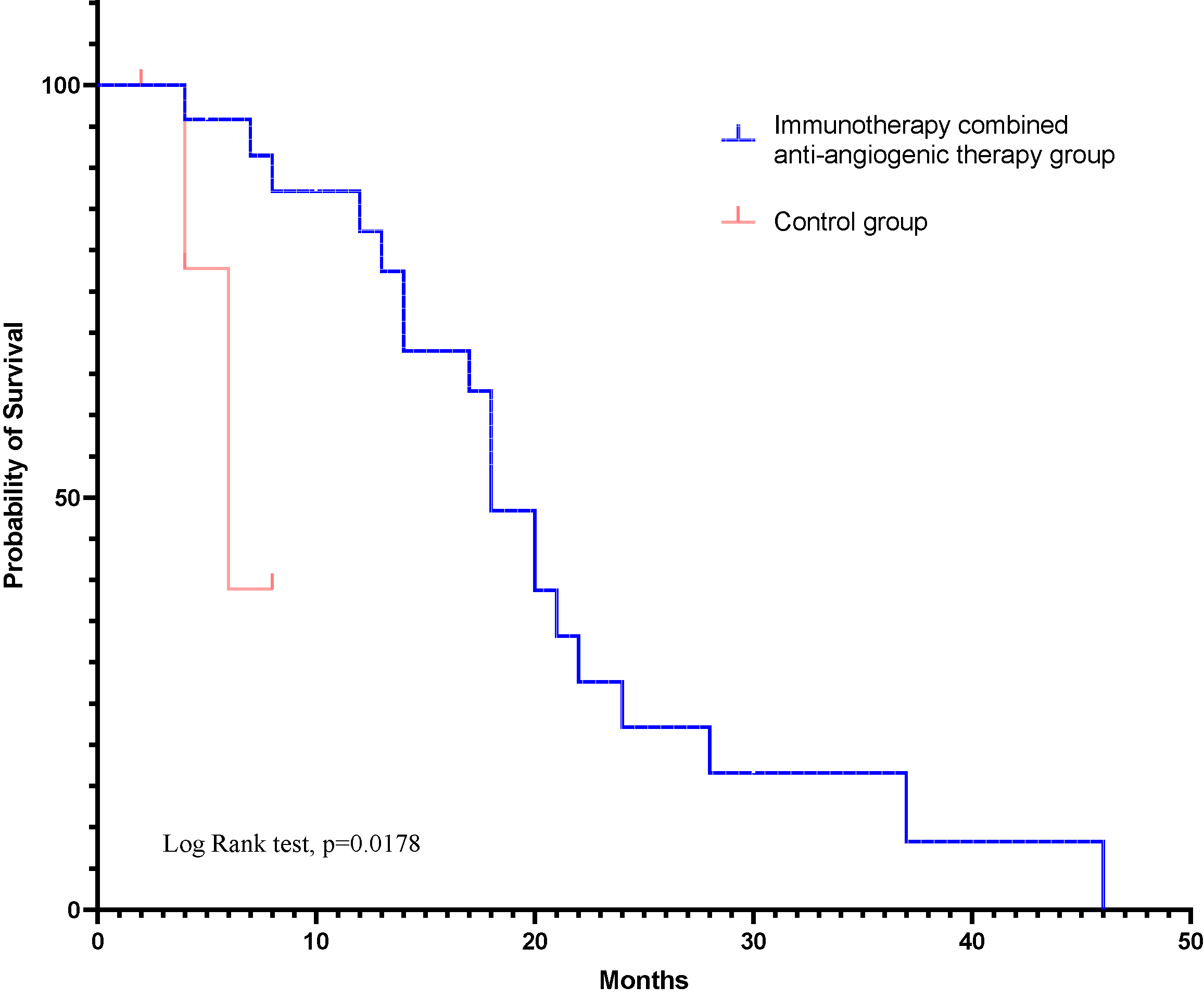

Supplement: Supplementary Figure 1 — Kaplan-Meier survival curves depicting progression-free survival for all patients in the immunotherapy combined with anti-angiogenic therapy group versus the control group (Log-Rank test, p < 0.05). [file Image1.tif]
